# Bacterial attachment and biofilm formation on surfaces are reduced by small-diameter nanoscale pores: how small is small enough?

**DOI:** 10.1038/npjbiofilms.2015.22

**Published:** 2015-12-02

**Authors:** Guoping Feng, Yifan Cheng, Shu-Yi Wang, Diana A Borca-Tasciuc, Randy W Worobo, Carmen I Moraru

**Affiliations:** 1 Department of Food Science, Cornell University, Ithaca, NY, USA; 2 Department of Mechanical, Aerospace and Nuclear Engineering, Rensselaer Polytechnic Institute, Troy, NY, USA

## Abstract

**Background/Objectives:**

Prevention of biofilm formation by bacteria is of critical importance to areas that directly affect human health and life including medicine, dentistry, food processing and water treatment. This work showcases an effective and affordable solution for reducing attachment and biofilm formation by several pathogenic bacteria commonly associated with foodborne illnesses and medical infections.

**Methods:**

Our approach exploits anodisation to create alumina surfaces with cylindrical nanopores with diameters ranging from 15 to 100 nm, perpendicular to the surface. The anodic surfaces were evaluated for attachment by *Escherichia coli*, *Listeria monocytogenes*, *Staphylococcus aureus* and *Staphylococcus epidermidis*. Cell–surface interaction forces were calculated and related to attachment.

**Results:**

We found that anodic alumina surfaces with pore diameters of 15 and 25 nm were able to effectively minimise bacterial attachment or biofilm formation by all the microorganisms tested. Using a predictive physicochemical approach on the basis of the extended Derjaguin and Landau, Verwey and Overbeek (XDLVO) theory, we attributed the observed effects largely to the repulsive forces, primarily electrostatic and acid–base forces, which were greatly enhanced by the large surface area originating from the high density, small-diameter pores. We also demonstrate how this predictive approach could be used to optimise different elements of surface topography, particularly pore diameter and density, for further enhancing the observed bacteria-repelling effects.

**Conclusions:**

We demonstrate that anodic nanoporous surfaces can effectively reduce bacterial attachment. These findings are expected to have immediate, far-reaching implications and commercial applications, primarily in health care and the food industry.

## Introduction

Biofilms are the prevailing lifestyle of bacteria in most natural environments. They consist of microbial communities that usually accumulate at solid–liquid interfaces and are entrapped in a matrix of highly hydrated extracellular polymeric substances.^1^ The quiescent lifestyle of microbial cells living in such a densely packed diffusion barrier is responsible for their high tolerance to environmental stresses.^2^ Cells in biofilms have been deemed 100 to 1,000 times more resistant to antibiotics and disinfecting agents than planktonic cells.^3,4^ Biofilm formation by pathogenic bacteria has deleterious, sometimes fatal consequences, and leads to severe contamination problems in medicine, dentistry, food processing, water treatment and other areas that directly affect human health and life. For instance, it is estimated that approximately 80% of all medical infections are derived from biofilm growth of pathogens.^5^ Biofilms formed by pathogens in food processing plants are a major culprit in the spread of foodborne diseases, which claim thousands of lives and amount to losses of about $78 billion/year in the United States alone.^6^

Most studies attempting to mitigate the effects of biofilms focus on interventions aimed to kill microbial cells in biofilms already present on solid surfaces.^7,8^ However, such strategies have limited efficacy owing to bacterial persistence and resistance in preformed biofilms.^9,10^ Surface modification is emerging as a promising strategy for preventing biofilm formation on abiotic surfaces. There is increasing evidence that bacterial attachment and subsequent biofilm formation are significantly impacted by surface topography.^11–13^ For surfaces with topographic features at the micrometric scale, comparable with the size of prokaryotic cells, cells tend to position themselves such that they maximise contact area with the surface, which favours attachment.^11,12^ Surfaces with topographic features of dimensions much smaller than microbial cells, in the submicrometric or nanometric range, have been reported to inhibit attachment by reducing the contact area between bacteria cells and the surface.^11,13^ In addition, surface topography at the nanoscale can create energetic situations unfavourable for bacterial attachment, and induce repulsive surface–bacteria interaction forces that impair attachment and subsequent biofilm formation.^14,15^ Although most nanostructuring methods available today require cleanroom technologies and are prohibitively expensive for large-scale applications, anodisation is an inexpensive, commercially available electrochemical method that allows relatively easy control of surface features in the nanometre range.^12^

A recent study by our research group showed that anodic alumina surfaces with nanoscale cylindrical pores of diameters smaller than 25 nm are able to reduce bacterial attachment by the non-pathogenic *Escherichia coli* and *Listeria innocua*.^15^ The observed effects were largely attributed to additional repulsive forces contributed by the large surface area of those substrates with a high density of small-diameter pores. The current report builds on these promising findings and tests the ability of anodic alumina surfaces to reduce bacterial attachment for several pathogenic strains. A quantitative prediction of bacteria–surface interaction forces is used to understand how to further optimise surface topographical features and physicochemical properties and thus create surfaces with a stronger ability to prevent bacteria attachment and biofilm formation.

As challenge organisms, four of the most feared pathogens associated with medical, biomedical or food processing environments were selected: *E. coli* O157:H7, *L. monocytogenes*, *Staphylococcus aureus* and *Staphylococcus epidermidis*, along with the non-pathogenic *E. coli* K12. *L. monocytogenes* can cause illness, death and abortion, and is of particular concern for immuno-compromised individuals and pregnant women. The United States Centers for Disease Control and Prevention estimates that about 2,500 listeriosis and 500 associated deaths occur yearly.^16^ The ubiquitous *L. monocytogenes* can be transmitted through raw foods, the environment, utensils or processing equipment.^17–19^ Infection with *E. coli* O157:H7 can lead to severe foodborne illness, specifically haemorrhagic diarrhoea and haemolytic uraemic syndrome.^16,20,21^ Contamination with *S. aureus* is the root cause for a range of illnesses, from food poisoning to infections of the skin and soft tissue, to respiratory, bone, joint and endovascular disorders; *S. aureus* is the most frequently isolated pathogen from wound infections.^22,23^ *S. epidermidis* has been associated with bacteremia, catheter-related infection, central nervous system shunt infection, endocarditis, urinary tract infection, surgical site infection and endophthalmitis.^24^

## Materials and Methods

### Surface fabrication

Nanoporous aluminium oxide (alumina) surfaces with pore diameters of 15, 25, 50 and 100 nm were prepared by two-step anodisation of high purity aluminium (99.99%, Alfa Aesar), which has been described in detail before.^15,25^ The aluminium substrate was first subjected to mechanical and electrochemical polishing, with an intermediate annealing process meant to release internal stresses. The polished substrate was immersed in an etchant to remove the thin alumina layer formed during electrochemical polishing. The first anodisation step was carried out at room temperature using a setup similar to that used for electrochemical polishing. The voltage and anodising mixture depended on the pore size. The first porous alumina layer was etched away and a second anodisation step was performed, during which pore growth was initiated from dents left over by the nanopores in the first layer, resulting in regular surface features.^25,26^ Nanosmooth alumina surfaces of 10×10×0.5 mm (Alfa Aesar, Ward Hill, MA, USA) were used as a control. The nanosmooth control used here had similar surface roughness (root-mean-square roughness *R*_rms_ <1 nm), but different surface properties compared with that used in our previous study^15^ (a water contact angle of 39.3±1.1° as compared with 67.5±5.0° previously).

### Bacteria attachment

Cultures of *E. coli* O157:H7 ATCC 43894, *E. coli* K12, *L. monocytogenes* 10403S, *S. aureus* 9144, *S. epidermidis* ATCC 35984 were maintained in tryptic soy broth with 20% (volume/volume) glycerol at −80 °C. Cultures were reactivated on tryptic soy agar at 37 °C for 24 h. They were grown in tryptic soy broth for 24 h and subcultured in tryptic soy broth for 16 h at 37 °C. The experimental procedure was described in detail before.^15^ Briefly, 16-hour-old cultures of planktonic cells at a diluted concentration of ~10^7^ CFU/ml were incubated statically for 48 h with vertically placed anodised alumina surfaces and the nanosmooth control, respectively, at the optimal growth temperature for each bacterial strain, then retrieved and evaluated for bacterial attachment. An incubation time of 48 h was chosen since it was previously observed that this time point allowed bacteria to attach to the surfaces in sufficient numbers for a meaningful quantitative assessment, but without significant biofilm formation.^15^ Surfaces were placed vertically to reflect true attachment and minimise the effect of cell sedimentation due to gravity. For modelling purposes, the nutritive broth was approximated as a 1:1 type electrolyte solution of ionic strength 0.1 M, at pH 7 and a temperature of 310 K (37 °C).

### Confocal laser scanning microscopy

The surfaces with attached cells were gently removed from the culture and rinsed in sterile saline solution (0.15 M NaCl), three times, to remove lightly attached cells. The bacterial biomass was labelled with Syto 9 (Molecular Probes Inc., Eugene, OR, USA). A Zeiss 710 confocal laser scanning microscopy equipped with inverted objectives was used to acquire three-dimensional images of live bacteria, as described before.^15^ For every type of surface, six replicates (two surfaces per each of three independent experiments) were used. On each sampled surface, at least five randomly selected and evenly spaced fields (338.4×338.4 μm^2^) were scanned. Three-dimensional images of biomass matrices were constructed using Volocity (version 5.2.1, PerkinElmer, Waltham, MA, USA).

### Biomass quantification

The total biomass and surface coverage were quantified using COMSTAT, a computer programme designed specifically for this purpose.^27^ A threshold value of 3 was assigned to all the individual image stacks. Quantified parameters were: biomass accumulation (μm^3^/μm^2^), obtained by dividing the overall biomass volume by the substratum area; and layer coverage, given by the percentage of the area occupied by bacteria in each optical layer.

### Scanning electron microscopy

Visualisation of biomass structures was conducted with a Zeiss LEO 1550 field emission scanning electron microscopy, and images acquired with the SmartSEM software (Carl Zeiss Microscopy, LLC, Hamburg, Germany). Surfaces were retrieved at 48 h and rinsed in saline solution to remove lightly attached cells. The biomass on the surfaces was fixed using 2.5% (w/v) glutaraldehyde in 0.05 M sodium cacodylate buffer at 4 °C for 2 h. Samples were then rinsed in cacodylate buffer three times, 5 min each time, and subjected to secondary fixation with 1% (w/v) osmium tetroxide in cacodylate buffer, for 1 h. The fixated samples were rinsed in cacodylate buffer three times, then dehydrated using gradient ethanol solutions of 25% (v/v), 50, 70, 95, 100 and 100%, for 10 min each, followed by critical point drying with carbon dioxide. Dried surfaces were mounted to scanning electron microscopy stubs and coated with evaporated carbon. A voltage of 1 to 5 kV was used, depending on the sample.

### Contact angle measurement

Contact angles of water, glycerol and diiodomethane on both bacterial cell lawns and alumina substrates were determined by the sessile drop method with a Rame-Hart 500 goniometer (Rame-Hart Inc., Succasunna, NJ, USA), as described before.^15^

### Surface electric charges

The zeta potential of the bacterial cells was measured using a Malvern Zetasizer nano-ZS with disposable folded capillary cells (Malvern Instruments, Malvern, UK), as described previously.^15^ Zeta potential of the alumina was taken from Li and Logan.^28^

### Statistical analysis

One-way analysis of variance and post-analysis-of-variance Tukey’s test were used to compare multiple means. All analyses were performed with JMP Pro 11 (SAS Institute, Cary, NJ, USA). The adjusted XDLVO model was constructed and computed using Mathematica 9.0 (Wolfram, Champaign, IL, USA).

## Results

### Biomass accumulation and structure on nanotopographic anodic alumina surfaces

**Figure 1:**
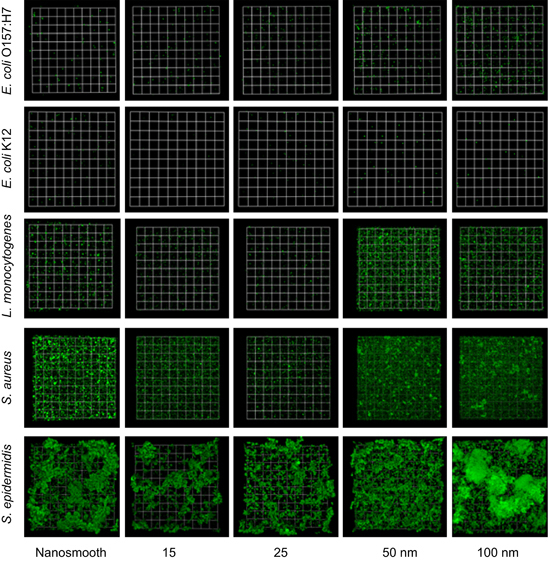
Constructed confocal laser scanning microscopy (CLSM) three-dimensional images of 48-hour-old biofilms of *E. coli* O157:H7, *E. coli* K12, *L. monocytogenes*, *S. aureus* and *S. epidermidis* on nanosmooth alumina (control) and anodised surfaces of 15, 25, 50 and 100 nm pore diameter. The presented images have biomass accumulation close to the average for their surface type, so that images are representative. Scale units (small grid) are 34 μm in length.

**Figure 2:**
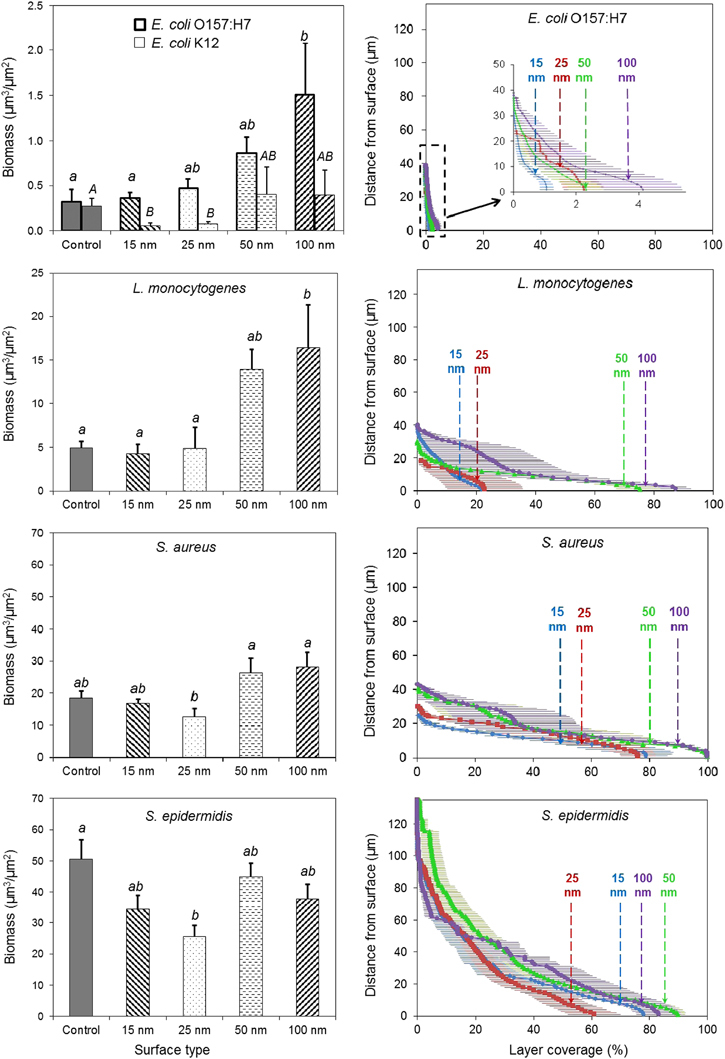
Differences in biomass accumulation over 48 h by *E. coli*, *L. monocytogenes, S. aureus* and *S. epidermidis* among alumina surfaces. Left panels: quantified average values of biomass (μm^3^/μm^2^) accumulated on nanosmooth alumina (control), and anodised surfaces of 15, 25, 50 and 100 nm pore diameter. Values not connected by the same letter are statistically different from each other (*P*<0.05). Error bars represent standard error of mean. Right panels: layer coverage as a function of distance from the surface. Error bars represent standard errors.

Consistent with our previous observations,^15^ presence of bacteria on the anodic surfaces with pores of 15 and 25 nm was largely reduced compared with the anodised surfaces with larger pore sizes, for all strains. Both visual observations (Figure 1) and the quantitative biomass evaluation (Figure 2) showed that the extent of biomass accumulation varied considerably among the tested microorganisms. For *E. coli* K12, *E. coli* O157:H7 and *L. monocytogenes*, a thin layer of uniformly distributed cells was observed on all surfaces, while for *S. aureus* and *S. epidermidis*, both prolific biofilm formers, biomass accumulation was much more significant, particularly on the surfaces with pore diameters above 50 nm (Figure 2, left panels). At 48 h, the biomass accumulation for *E. coli* O157:H7 on the nanosmooth, 15, 25, 50 and 100 nm surfaces was 0.32 μm^3^/μm^2^, 0.36 μm^3^/μm^2^, 0.47 μm^3^/μm^2^, 0.86 μm^3^/μm^2^ and 1.51 μm^3^/μm^2^, respectively. The biomass accumulation for *E. coli* K12 was slightly lower than for *E. coli* O157:H7. For *L. monocytogenes*, the biomass accumulation was about one order of magnitude higher than for the *E. coli* strains, ranging from 4.27 μm^3^/μm^2^ on 15 nm surfaces to 16.40 μm^3^/μm^2^ on 100 nm surfaces. Biomass accumulation by *S. aureus* and *S. epidermidis* followed a similar trend, but was much more pronounced compared with the two *E. coli* strains and *L. monocytogenes*. *S. aureus* had the lowest biomass accumulation on the 15 nm surface (16.91 μm^3^/μm^2^) and the highest on the 100 nm surface (28.19 μm^3^/μm^2^). *S. epidermidis* had the lowest biomass on the 25 nm surface (25.54 μm^3^/μm^2^) and the highest on the nanosmooth control (50.56 μm^3^/μm^2^). The thickness of the *S. epidermidis* biomass often exceeded 100 μm, several times higher than for any of the other strains tested. For the 100 nm surfaces, 48-h biomass accumulation by *S. epidermidis* had the clear traits of a dense biofilm and often included microcolonies (Figure 1, bottom right). As a note, the nanosmooth controls used in the current work were different than those used in our previous study.^15^ This resulted in slightly different levels of biomass accumulation than previously reported for the nanosmooth alumina controls, but the attachment trends were maintained.

Overall, the trend in biomass accumulation on the anodic alumina surfaces by all strains agrees with our previous observations.^15^ Owing to some variability in the data, not all differences in biomass accumulation among surface types were statistically significant (*P*<0.05). Nonetheless, a closer analysis of the biomass structure further substantiates the differences among surface types. We generated vertical three-dimensional confocal laser scanning microscopy scans of the biomass structure for all anodic alumina surfaces, in 1 μm vertical increments, and calculated the surface coverage by biofilm for each layer. The right panels in Figure 2 show surface coverage at different distances from the substratum for the four pathogenic strains. For all strains, biofilm surface coverage was lowest for the 15 and 25 nm surfaces, and highest for the 50 and 100 nm surfaces. For the weak biofilm former *E. coli* O157:H7, maximum coverage ranged between 1% on the 15 nm surfaces and 4% on the 100 nm surfaces. The most striking differences in coverage among surfaces were observed for *L. monocytogenes*, for which maximum coverage on the surfaces with small pore sizes (about 20%) was four-fold smaller than on surfaces with larger pore sizes (about 80%). The prolific biofilm former *S. epidermidis* had high coverage on all surfaces, ranging from a low 61% on the 25 nm surfaces to a high 90% on the 50 nm surfaces. For the other strains, significant coverage was found mostly within a few micrometres from the surface, whereas for *S. epidermidis,* biomass coverage was very high, even tens of micrometres from the surface.

**Figure 3:**
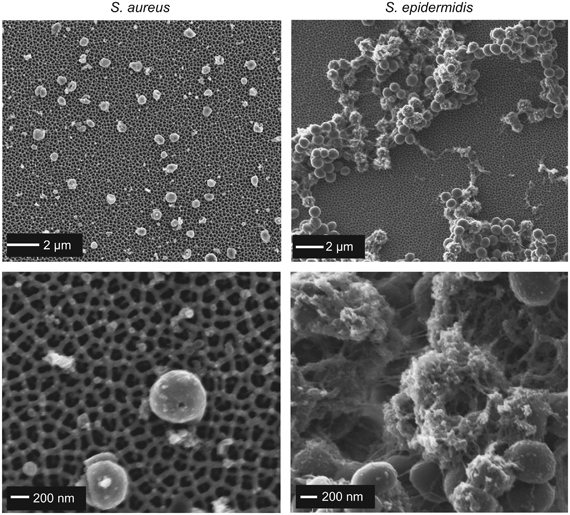
Scanning electron microscopy images of *S. aureus* (left images) and *S. epidermidis* cells (right images) at low magnification (upper) and high magnification (lower) after 48 h contact time with anodic alumina surfaces with 100 nm pore diameter. Bottom right image shows *S. epidermidis* cells entrapped in a matrix of extracellular material.

To further investigate the biomass structure, we conducted scanning electron microscopy visualisation of the surfaces with the largest pore size (100 nm), which had either the largest or second largest biomass accumulation. It should be noted that the scanning electron microscopy images cannot be used for quantitative purposes, since some cell detachment from the surfaces may have occurred during scanning electron microscopy sample preparation. Figure 3 shows scanning electron microscopy micrographs for *S. aureus* (left) and *S. epidermidis* (right). For *S. aureus*, a single layer of cells was generally observed, with minimal amount of extracellular material. *S. epidermidis* on the other hand showed substantial, vertically grown biofilms, with a high density of bacterial cells tightly intertwined in a matrix of extracellular material, which is very typical of *S. epidermidis* biofilms.^30^ The tremendous biofilm forming ability of *S. epidermidis* has been attributed to a large extent to the contribution of specific biological factors, mainly the polysaccharide intercellular adhesin encoded by the intercellular adhesion (*ica*) locus.^31,32^ All other bacterial strains showed a single layer of cells, and no significant indication of extracellular substances (images not shown). On the basis of this corroborated evidence, in the subsequent discussion, we will refer to the biomass accumulation as attached cells for all the strains except *S. epidermidis*, for which a biofilm was formed.

Overall, this study clearly shows that the small nanoscale pore anodic alumina surfaces can effectively limit cell attachment and biofilm formation by a range of bacteria relevant for medical, biomedical and food processing applications. The trend was similar regardless of Gram-positive or Gram-negative status, rod or coccus shape, or the ability of the cells to express appendages under the conditions tested.

### Bacterial attachment correlates with surface–bacteria interaction forces

**Figure 4:**
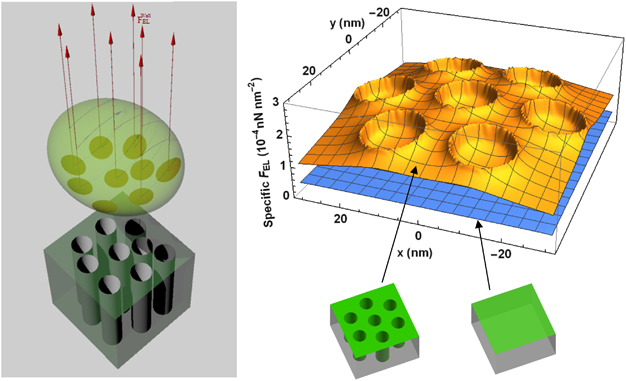
Electrostatic repelling force field exerted on bacterial cells by the nanoporous surface. Left: schematic representation of the electrostatic repelling forces exerted on a bacterial cell by the nanopores located directly underneath the cell. Right: spatial distribution of the electrostatic repulsive force field exerted on *E. coli* O157:H7 cells by an anodic alumina surface with cylindrical pores of 15 nm diameter and 2,259 nm pore depth (top plot) compared with a smooth alumina surface (bottom plot), at a cell–surface separation distance of 0.2 nm. The surface components contributing to the force fields are illustrated in green.

Previously, we found strong evidence of a correlation between attachment by two bacteria strains and the overall cell–surface interaction force, calculated using the extended Derjaguin and Landau, Verwey and Overbeek (XDLVO) theory.^15^ One important prediction made using this model was the maximum repulsive force that a bacterial cell needs to overcome and come into direct contact with a surface, termed ‘*F*_max_’. We were able to show that as *F*_max_ increased, the number of bacteria attached to a surface decreased.^15^ Therefore, the XDLVO predictive approach was used to estimate bacteria–surface interaction forces for the strains tested in this study. Predictions were limited to those strains that did not show biofilm formation over the 48 h duration of the test: *E. coli* O157:H7, *E. coli* K12, *L. monocytogenes* and *S. aureus. S. epidermidis* was not included in these predictions, because the presence of a significant amount of extracellular material did not allow an accurate estimation of the number of cells (Figure 4).

Cell properties required by the model were determined experimentally as described in the Materials and Methods section, and their measured values are summarised in Supplementary Table S1. When applying the XDLVO model, the following simplifying assumptions were made: (i) all cells were assumed to be spherical in shape, and for rod-shaped bacteria (*E. coli* and *Listeria*) an equivalent radius was calculated; (ii) surfaces have a fully wetting (Wenzel) behaviour^33^ and (iii) each surface was assumed to be an infinite planar surface relative to a bacterial cell. The surface properties are included in Supplementary Table S2, and the other constants used in the calculations are in Supplementary Table S3.

The overall interaction force between a bacterium cell and the anodic alumina surfaces, $F_{\mathrm{Total}}^{\mathrm{XDLVO}}$, was calculated by the vector addition of three force components: electrostatic force (*F*_EL_), acid–base interaction force (*F*_AB_), and Lifshitz-van der Waals interaction force (*F*_LW_). As all the forces are affected by the nanopores underneath the cells, they were adjusted to take into consideration this effect (denoted as ‘Adj’):

(1)FTotalXDLVO=FLWAdj+FABAdj+FELAdj

The full expression of equation (1) and the derivation of its components are presented in detail in our previous study.^15^ Briefly, the Lifshitz-van der Waals interaction force between the cells and the surfaces $\left(F_{\mathrm{LW}}^{\mathrm{Adj}}\right)$ was calculated using the retarded Hamaker expression. The acid–base interaction force $\left(F_{\mathrm{AB}}^{\mathrm{Adj}}\right)$, which incorporated the effect of surface energy, was calculated using the extended Young equation.^34^ As acid–base interactions are short range interactions, it was considered that only the top rim of the vertical surface of the cylindrical nanopores (2 nm from the surface) effectively contributes to the acid–base interaction. This portion of the internal surface of the cylindrical pores was approximated as a ring of hemispheres distributed uniformly along the circumference. The repulsion force exerted on one bacterium by the total number of cylindrical walls underneath that cell was calculated considering the pore diameter and surface porosity, as well as the radius of the effective circular interaction area for each type of bacteria.

The electrostatic interaction force $\left(F_{\mathrm{EL}}^{\mathrm{Adj}}\right)$ was calculated for each pore and its surrounding area and the total force was determined by multiplying the value of this force by the number of pores underneath one bacterium cell. An example of the contribution of nanoscale topographical features to the magnitude and spatial distribution of interaction forces, the specific *F*_EL_ (electrostatic force per unit area, or ‘electrostatic pressure’) exerted on a bacterial cell by the surface, plotted as a function of the radial distance from the centre of a cylindrical pore, is shown in Supplementary Figure S1. Regardless of pore size, the vertical walls of the cylindrical pores from the porous anodic surfaces contributed to an increase in the electrostatic force compared with that generated by a smooth surface of similar chemistry. Figure 4 shows both a schematic representation of the total electrostatic forces acting on a *E. coli* O157:H7 cell (left), and the field of electrostatic forces contributed by a surface area equivalent to a hexagonal array of 15 nm pores (right), at a cell–surface separation distance where electrostatic forces are significant for this bacterial strain. The force field plot on the right clearly shows that the repulsion exerted by the 15 nm surface greatly exceed the repulsion by the nanosmooth control surface for *E. coli* O157:H7, which is consistent with the biomass accumulation shown in Figure 1 and Figure 2. Similar plots can be generated for the other forces.

The total cell–surface interaction force was then calculated as a function of the cell–surface separation distance (Supplementary Figure S2). In close proximity of the surface, ranging from fractions of a nanometre to several nanometres, depending on the bacterial strain, the bacterium–surface interaction force was attractive for all surface–strain pairs, owing to the short range attractive Lifshitz-van der Waals forces. At separation distances beyond a few nanometres, the repulsive electrostatic and acid–base forces become dominant for all the strains. For the anodic surfaces with the smallest pores, this contribution is particularly significant owing to the large number of cylindrical pores per surface area. Consequently, the total repelling force $\left(F_{\mathrm{Total}}^{\mathrm{XDLVO}}\right)$ is particularly high for the 15 nm and 25 nm pore surfaces, which have a high density of vertical pores per unit surface area.

**Figure 5:**
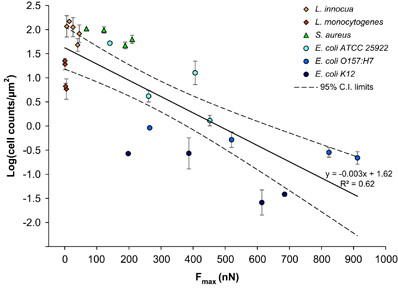
Bacteria cell counts in the biofilm per unit area of surface as a function of *F*_max_, after 48 h of incubation. The regression equation and *R* ^2^ value are for all the strains pooled together. Error bars represent standard errors. C.I., confidence interval.

*F*_max_ for all bacteria–anodic surface pairs used in the study was plotted against the cell counts (Figure 5), calculated on the basis of the biomass volume and the measured cell size (Supplementary Table S1). The data points for two non-pathogenic strains (*L. innocua* and *E. coli* ATCC 25922) from our previous work^15^ were also included in this analysis. This plot allowed several important observations. First, for all the strains, the greater the magnitude of the energy barrier, represented by the repulsive *F*_max_, the fewer cells accumulated on the surface. A linear correlation between *F*_max_ and the number of attached cells was obtained for each strain. As only four data points per strain were available, and because of the variability of some data points, not all correlations were statistically significant. For the strains tested in this study, the values for the coefficient of determination were as follows: *R* ^2^=0.743 for *S. aureus*, *R* ^2^=0.864 for *L. monocytogenes*, *R* ^2^=0.815 for *E. coli* K12 and *R* ^2^=0.998 for *E. coli* O157:H7. Figure 5 also illustrates a clustering of bacteria from different species. The two *Listeria* strains (diamond symbols) showed the strongest dependence on *F*_max_, which means that small increases in the repulsive force can be extremely effective in reducing the attachment by these bacteria to the anodic surfaces. The three *E. coli* strains (circle symbols), while showing a good correlation between attachment and *F*_max_, were less sensitive to the magnitude of *F*_max_ compared with *Listeria. S. aureus* (triangle symbols) had a behaviour intermediate between *Listeria* and *E. coli*. The similar response to *F*_max_ of bacteria from the same species is not only indicative of the role of biological factors in attachment, but also shows that the physicochemical approach used here is able to reflect these differences.

The other very important observation is that a statistically significant (*P*<0.001) linear correlation between *F*_max_ and the number of attached cells from all bacterial strains was obtained. The solid line in Figure 5 represents the linear regression line, and the dotted lines represent the 95% confidence interval range for the linear regression. The confidence interval accounts for both the variance of the experimental data and the uncertainty in the numerical coefficients of the linear regression. The 95% confidence interval lines are the closest in the middle of the *F*_max_ range, and further apart towards the ends of the *F*_max_ range, which indicates that the uncertainty of the prediction increases for both very low and very high attachment.

## Discussion

Bacterial attachment to abiotic surfaces and subsequent biofilm formation are complex processes, controlled by the interplay between biological factors, such as secretion of extracellular materials by the bacteria,^29^ bacterial appendages and other cell surface structures that can contribute to bacterial sensing of the surface,^9^ and physicochemical factors, such as surface topography, surface charge and surface energy.^14^ As it is practically impossible to alter the properties of naturally occurring bacteria and make them less likely to attach to abiotic surfaces, the more feasible approach to tackle biofouling is to reduce the propensity of the abiotic surfaces to bacterial attachment, by altering their surface chemistry and topography.

The correlation between bacteria attachment and surface topography has been investigated for a long time. Commonly used surface roughness parameters such as average and root-mean-square roughness describe only the height variation of the surface, but not the spatial distribution or the shape of surface features.^35^ Donoso *et al*.^36^ demonstrated that neither parameter is a reliable predictor for the interfacial area from which surface-based forces (i.e., *F*_AB_ and *F*_EL_) arise, as the relationship between these parameters and interfacial area is not monotonic. These results explain at least, in part, why despite the volume of work in this area, the mechanisms by which surface roughness and topography modulate attachment remain largely unclear, especially at a scale smaller than the dimension of a bacterial cell.^28,35^

The present study shows that attachment by *E. coli* O157:H7, *E. coli* K12, *L. monocytogenes*, *S. aureus* and *S. epidermidis* was impaired on anodic alumina surfaces with pore sizes of 15 and 25 nm in diameter as compared with surfaces with larger pores, and in some cases with the nanosmooth control surfaces (Figure 1). The visual confocal laser scanning microscopy observations were validated by quantitative assessment of the biofilm matrices (Figure 2).

This study also allowed us to make several very useful observations regarding the effect of abiotic surface properties on bacterial attachment. To date, many biofouling studies used surface energy values calculated from apparent contact angles as a predictor of attachment. Several studies suggest that highly hydrophobic surfaces, which have water contact angles higher than 90°, have a lower propensity for microbial attachment and biofouling.^37^ Nonetheless, the conventional hydrophobic–hydrophilic dichotomic categorisation of surfaces and bacterial cells has been proved insufficient to make accurate predictions about bacterial attachment. Hook *et al*.^38^ found no correlation between bacterial attachment and water contact angles for 496 polymeric materials. Rather, they suggested that surface chemical groups dictated the propensity of bacterial attachment onto polymer surfaces rather than water contact angle alone. Our results also show that neither hydrophobicity nor chemistry alone can be used as a predictor for attachment. Despite the fact that all porous surfaces used in our study had similar chemistry, the measured contact angles of three probe liquids varied notably among the surfaces (Supplementary Figure S3). Furthermore, the surfaces that were most effective against bacterial attachment had the smallest contact angles in all the three liquids and a clear hydrophilic behaviour, whereas surfaces with the largest pores were more hydrophobic, yet allowed higher attachment by bacteria. To understand the change in contact angle with pore size, it is important to consider the fundamental factors that dictate the apparent contact angles, namely the intrinsic (Young’s) contact angle of a liquid droplet on an ideal (rigid, flat, chemically homogeneous, insoluble, nonreactive) solid surface, and surface topography, which can enhance both hydrophobic and hydrophilic reactions.^39,40^ The trends in Supplementary Figure S3 agree with the Wenzel relation, which dictates that surface topography on hydrophilic surfaces (water contact angle <90°) enhances their apparent hydrophilicity.^40^ For alumina surfaces under consideration here, this was likely owing to the capillary effects caused by the cylindrical pores.^41^

A quantitative prediction of the propensity of bacteria to attach onto surfaces is not straightforward. In this work, such a prediction was obtained by using the comprehensive XDLVO model, which considers the role of cell–surface physicochemical interaction forces in attachment. This model has been used before, with varied degrees of success, in describing the interaction between colloidal particles and patterned surfaces,^42,43^ or the interaction between microbial cells and surfaces.^44–46^ One element lacking in previous applications of this model is that contributions from surface elements perpendicular to the surface were not considered. Taking this into account allowed us to explain the extraordinary bacteria-repelling effect of the 15 and 25 nm alumina surfaces. Specifically, this effect is attributed to the vertical sidewalls of the densely distributed cylindrical pores, which exerted additional electrostatic repulsion and acid–base repulsive forces on the bacterial cells found in the proximity of the surfaces.

The fact that the correlation between cell–surface interaction force, represented by *F*_*max*_, and attachment holds across several bacteria strains (Figure 5) is of considerable importance in that, despite the different cell wall structure, shape, presence or absence of cellular appendages, the adjusted XDLVO model is capable of predicting the trends in bacteria attachment with reasonable accuracy. This is also very relevant from a practical perspective. Although this study has been conducted on individual strains, in both natural and man-made environments, bacteria are present in multi-microbial communities. The overall correlation between *F*_max_ and attachment found here indicates that an increase in the repulsive force will be able to reduce attachment by multiple bacteria, albeit to different degrees.

**Figure 6:**
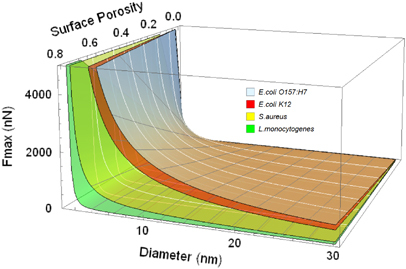
Predicted values of the maximum repelling cell–surface interaction force as a function of pore diameter and surface porosity of the alumina anodic surfaces for *E. coli* O157:H7, *E. coli* K12, *S. aureus* and *L. monocytogenes.*

As an example of how this model can be used as a design tool, we generated predictions of changes in cell–surface interaction forces induced by changes in the elements of surface topography. Our calculations show that pore diameter has a tremendous effect on the repulsive forces, with *F*_max_ increasing exponentially as pore diameter decreases. Increasing surface porosity at a fixed pore diameter results in a proportional increase in *F*_max_, while pore depth does not seem to have a significant effect beyond several tens of nanometres. Figure 6 illustrates how changing pore diameter and surface porosity affects *F*_max_ for all bacteria strains used in this study, except *S. epidermidis*. What is remarkable is that the effect of surface topography is similar for all microorganisms, even if the magnitude of *F*_max_ varies among the different strains. This is extremely meaningful from a practical perspective, because it indicates that further decreasing the pore size and increasing the surface porosity will improve the anti-attachment ability of anodic surfaces.

The findings of this study are of high importance, as they demonstrate a science based, yet relatively simple and practical way to prevent attachment and subsequent biofilm formation by diverse pathogenic, as well as non-pathogenic bacteria.
