# Supplementary figures and images for: Bacterial attachment and biofilm formation on surfaces are reduced by small-diameter nanoscale pores: how small is small enough?
Source: NPJ Biofilms Microbiomes. 2015 Dec 2;1:15022–. doi: 10.1038/npjbiofilms.2015.22 (PMC5515209; doi:10.1038/npjbiofilms.2015.22)

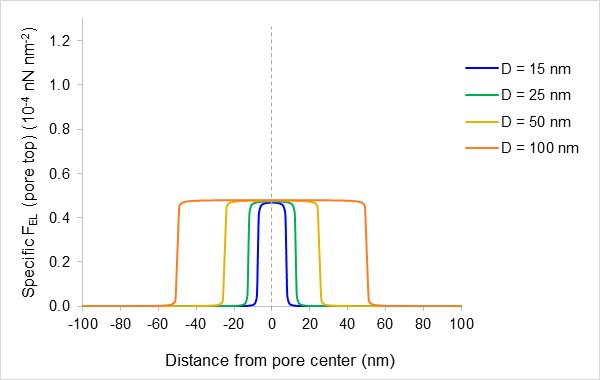

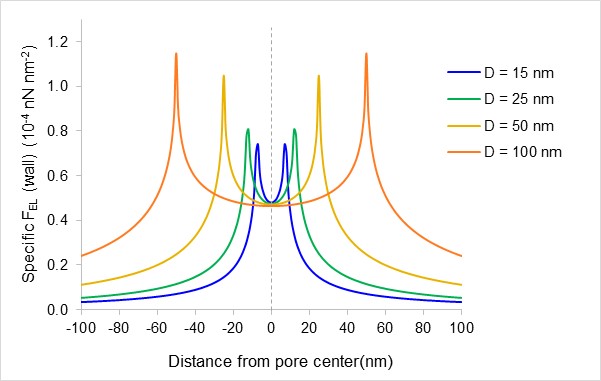

Supplement: Supplementary Figure S1 [file npjbiofilms201522-s1.doc]

| a) 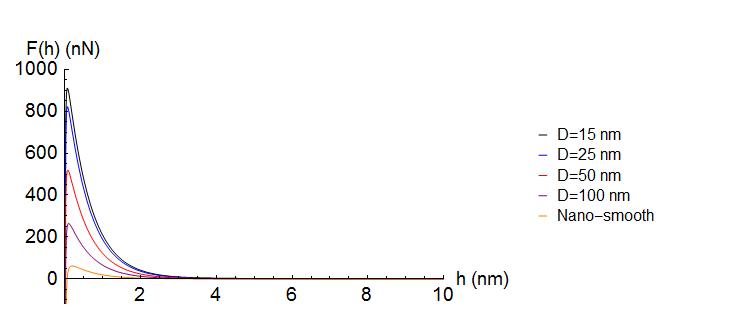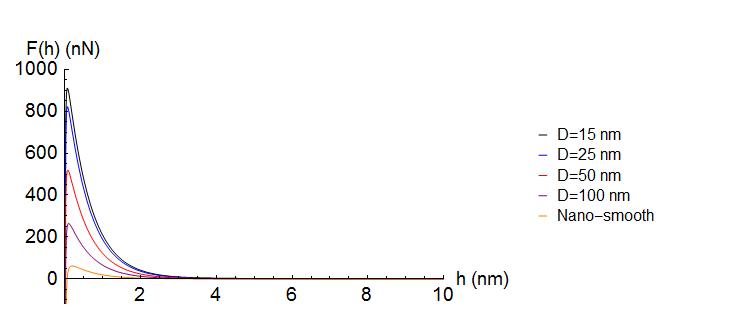 | b) 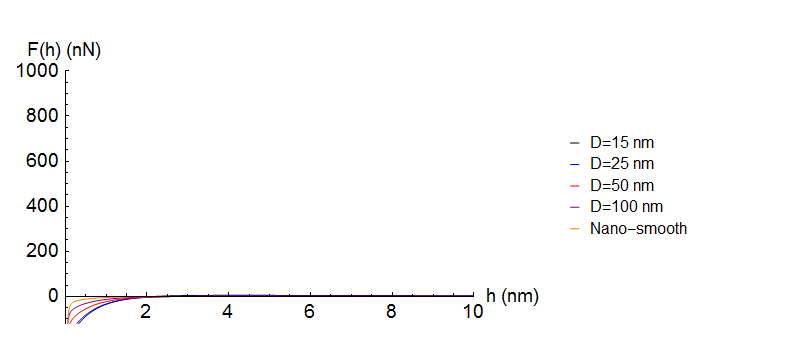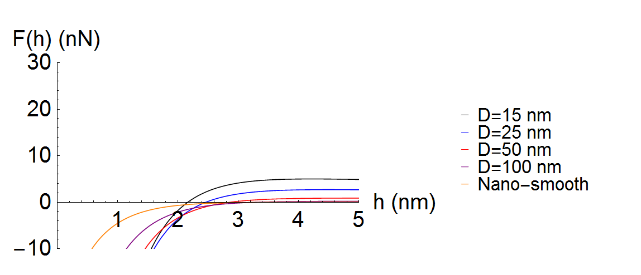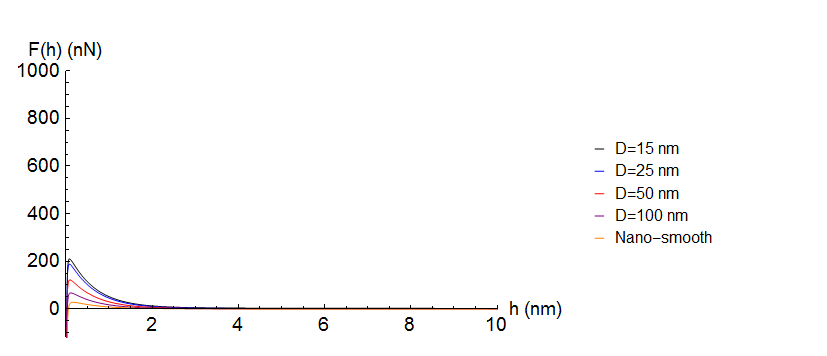 *f*7:H7 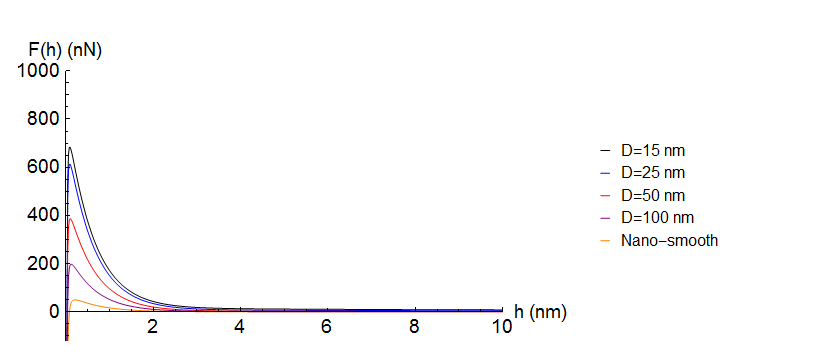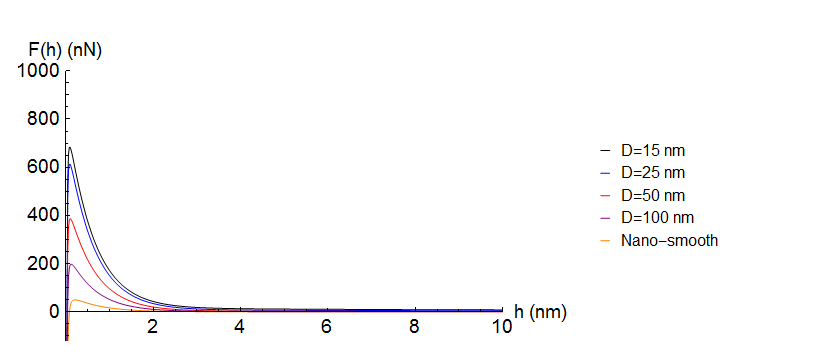 |
| --- | --- |
| c) 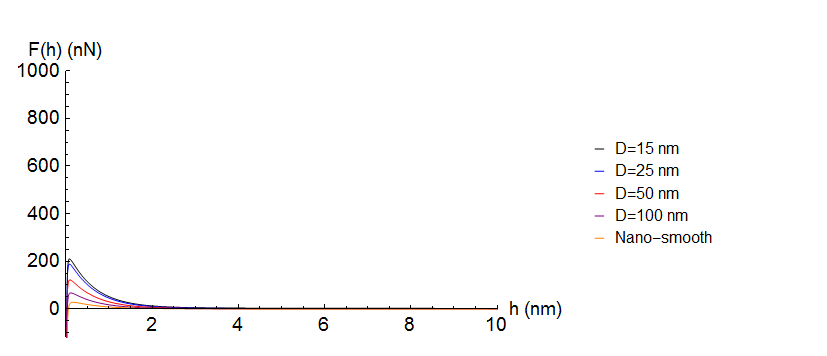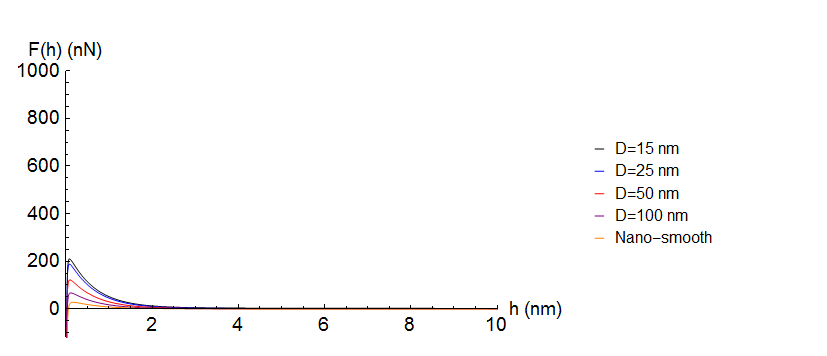 | d)  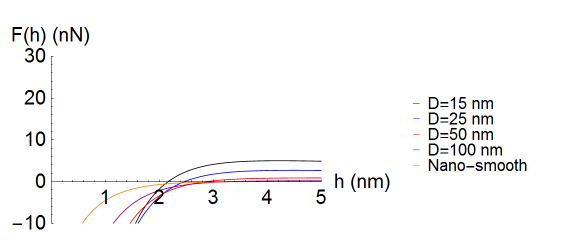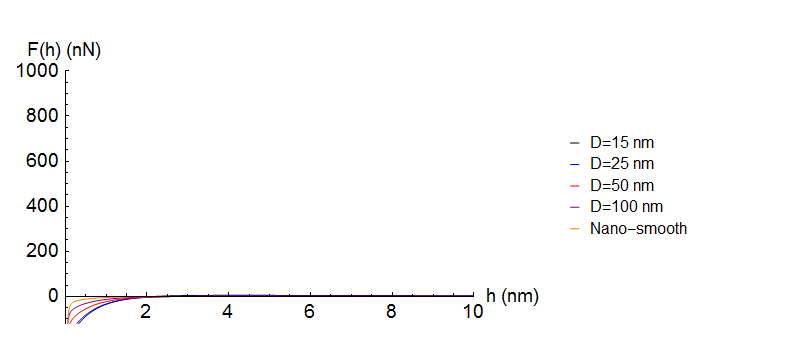 |

Supplement: Supplementary Figure S2 [file npjbiofilms201522-s2.doc]

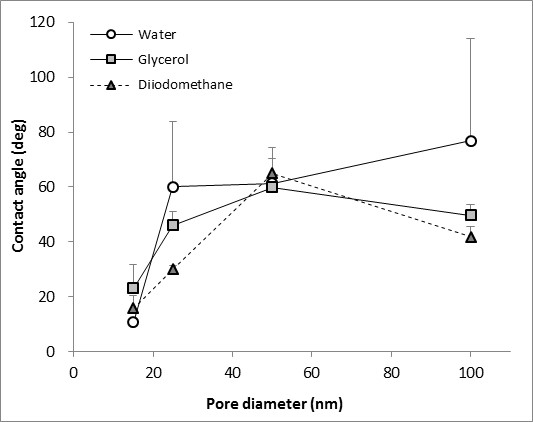

Supplement: Supplementary Figure S3 [file npjbiofilms201522-s3.doc]
