# Supplementary material for: Bacterial attachment and biofilm formation on surfaces are reduced by small-diameter nanoscale pores: how small is small enough?
Source: NPJ Biofilms Microbiomes. 2015 Dec 2;1:15022–. doi: 10.1038/npjbiofilms.2015.22 (PMC5515209; doi:10.1038/npjbiofilms.2015.22)
Supplement: Supplementary Information [file npjbiofilms201522-s4.doc]

**Bacterial attachment and biofilm formation on surfaces are prevented by small diameter nanoscale pores: how small is small enough?**

Guoping Feng^a^, Yifan Cheng^a^, Shu-Yi Wang^b^ Diana A. Borca-Tasciuc^b^*, Randy W. Worobo^a^, and Carmen I. Moraru^a^*

*^a^ Department of Food Science, Cornell University, Ithaca, NY 14853 USA;*

*^b^ Department of Mechanical, Aerospace and Nuclear Engineering, Rensselaer Polytechnic Institute, Troy, NY 12180 USA*

**Supplemental materials**

**Table S1. Physicochemical parameters used in the XDLVO model calculations for all bacterial strains tested**

| Bacterial strain | Contact angles (°) in various liquids | | | Zeta potential (mV) | Cell size | |
| --- | --- | --- | --- | --- | --- | --- |
|  | WCA | DCA | GCA |  | Volume (μm^3^) | Equivalent radius (nm) |
| *L. innocua* | 46.5±1.2 | 65.9±2.3 | 46.4±1.7 | -21.5±0.5 | 0.13 | 318 |
| *L. monocytogenes* | 56.7±0.5 | 50.4±1.2 | 70.1±0.9 | -21.1±0.5 | 0.73 | 559 |
| *E. coli* O157:H7 | 25.5±1.5 | 46.1±0.5 | 62.4±1.5 | -4.8±0.4 | 1.65 | 733 |
| *E. coli* K12 | 41.1±1.0 | 47.2±2.8 | 71.5±1.4 | -29.0±0.1 | 1.48 | 707 |
| *E. coli* ATCC 25922 | 41.4±0.5 | 55.6±3.4 | 75.7±2.0 | -22.4±0.3 | 0.50 | 494 |
| *S. aureus* | 47.0±1.9 | 60.0±3.5 | 76.4±0.9 | -23.4±0.4 | 0.27 | 399 |
| *S. epidermidis* | 15.8±1.4 | 62.1±0.5 | 91.3±1.1 | -16.3±0.4 | 0.20 | 361 |

**Table S2.** Properties of the alumina surfaces used in the study

1. Nanosmooth alumina (control surface)

| **Surface** | **Contact angles (°) in various liquids ^a^** | | | **Zeta potential (mV) ^b^** |
| --- | --- | --- | --- | --- |
|  | **WCA**  **(water)** | **DCA**  **(diiodomethane)** | **GCA**  **(glycerol)** |  |
| Nano-smooth alumina | 67.5±5.0 | 43.7±5.4 | 56.9±1.2 | -26.2±0.5 |

^a, b^ The contact angles and surface zeta potential measured on nano-smooth alumina were assumed to be the intrinsic values for the porous anodic alumina surfaces (i.e. the values for the infinitesimally small surface elements, prior to considering topographical effect imparted by the pores).

1. Anodic alumina

| **Anodic Alumina Type (Pore Size)** | **Porosity*** | **Pore Depth* (nm)** |
| --- | --- | --- |
| 15 nm | 0.176 | 2559 |
| 25 nm | 0.260 | 875 |
| 50 nm | 0.310 | 1189 |
| 100 nm | 0.306 | 1406 |

* Measured values

**Table S3.** Values of the constants used for calculating bacteria-surface interaction forces

| **Symbols** | **Parameters / constants** | **Values** | **Source** |
| --- | --- | --- | --- |
| 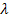 | Characteristic wavelength of bacteria-surface interaction | 100 nm | Gregory 1981 ^1^ |
| 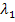 | Characteristic decay of acid-base interactions in water | 0.6 nm | Bhattacharjee *et al.* 1996 ^2^ |
| 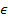 | Permittivity of water | 80×8.8854×10^-12^ (C^2^ J^-1^ m^-1^) |  |
| 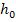 | Minimum separation between two bodies | 0.158 nm | Bhattacharjee *et al.* 1996 ^2^ |
| 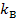 | Boltzmann constant | 1.38×10^23^ (J K^-1^) |  |
| 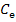 | The elementary charge | 1.602×10^-19^ C |  |
| *γ * | Surface tension of water, glycerol, diiodomethane | *(multiple values)* | Li *et al.* 2004 ^3^ |

**REFERENCES**

1 Gregory J. Approximate expression for retarded van dar Waals interaction. *J Colloid Interface Sci* 1981; **83**: 138–145.

2 Bhattacharjee S, Sharma A, Bhattacharya PK. Estimation and influence of long range solute. membrane interactions in ultrafiltration. *Ind Eng Chem Res* 1996; **35**: 3108–3121.

3 Li B, Logan BE. Bacterial adhesion to glass and metal-oxide surfaces. *Colloids Surf B Biointerfaces* 2004; **36**: 81–90.

**Figure Legends:**

**Figure S1.** Electrostatic repelling force exerted on *E. coli* O157:H7 cells by a circular portion of alumina of varying diameters (left), and the vertical walls (right) of cylindrical pores of different diameters, as a function of distance from the center of the pores, at a cell-surface separation distance of 0.2 nm.

**Figure S2.** Example of total cell-surface interaction force as a function of distance from the surface for one a) *E. coli* O157:H7 cell; b) *E. coli* K12; c) *S. aureus*; d) *L. monocytogenes* - the inset shows a zoomed-in plot.
**Figure S3.** Measured contact angles in water, glycerol, and diiodomethane for anodic alumina surfaces, as a function of pore diameter
